# A prospective, double-blinded cohort study using quantitative fetal fibronectin testing in symptomatic women for the prediction of spontaneous preterm delivery

**DOI:** 10.1186/s12884-023-05543-3

**Published:** 2023-04-04

**Authors:** Vivian Wai Yan Ng, Mimi Tin Yan Seto, Holly Lewis, Ka Wang Cheung

**Affiliations:** Department of Obstetrics and Gynaecology, Queen Mary Hospital, The University of Hong Kong, 102 Pokfulam Road, High West, Hong Kong

**Keywords:** Preterm delivery, Fetal fibronectin, Preterm labour

## Abstract

**Background:**

Spontaneous preterm birth (PTB) affects 6.5% of deliveries in Hong Kong. Quantitative fetal fibronectin (fFN) is under-utilised as a test for PTB prediction in Hong Kong. Our objective was to evaluate the effectiveness of quantitative fFN in predicting spontaneous PTB in women with symptoms of threatened preterm labour (TPTL) in our population.

**Methods:**

A prospective, double-blinded cohort study of women with a singleton gestation and TPTL symptoms presenting to a tertiary hospital in Hong Kong between 24 + 0 to 33 + 6 weeks was performed from 1st October 2020 and 31st October 2021. Women with vaginal bleeding, ruptured membranes, and cervical dilation > 3 cm were excluded. The primary outcome was to test the characteristics of quantitative fFN in predicting spontaneous PTB < 37 weeks. Secondary outcome was to investigate the relationship between fFN value and time to PTB. Test characteristics of quantitative fFN at different thresholds were evaluated.

**Results:**

48 women with TPTL were recruited. All had fFN testing at admission with the results being concealed from the obstetrician managing the patient. 10 mothers had PTB (< 37 weeks’ gestation). 7/48 (15%) had a subsequent PTB within 14 days from testing and 5 (10%) delivered within 48 h. The negative predictive value (NPV) of predicting delivery within 14 days was 97.3% and 100% when using a cut-off of < 50ng/ml and < 10ng/ml respectively. Using > 200 ng/ml as cut-off can also reliably predict delivery within 48 h – 7 days with positive predictive value PPV of 100%; as well as PTB before 37 weeks.

**Conclusions:**

Quantitative fFN has predictive value for spontaneous PTB prediction in symptomatic women in a Hong Kong population. fFN concentration could help clinicians rule out PTB and avoid unnecessary interventions and hospitalisation.

## Background

Preterm birth (PTB), defined as delivery prior to 37 weeks gestation is a major global health issue, affecting 15 million births worldwide [1]. Spontaneous PTB occurs in 6.5% of deliveries in Hong Kong [2]. The ability to predict PTB is challenging as approximately 50% of patients with TPTL subsequently deliver at term. This results in a significant number of women with receiving unnecessary treatment [2, 3]. Accurate identification of women in true PTB could allow timely interventions, such as antenatal corticosteroid therapy, magnesium sulphate for neuroprotection and transfer to a facility with appropriate level of neonatal care. It could also prevent unnecessary hospitalisation in women with low risk of PTB, thus reducing the associated social and economic costs [4].

Traditionally the diagnosis of preterm labour is made by regular uterine contractions with cervical effacement or dilatation at preterm gestation Studies have shown that symptomatic women with a shortened cervix (< 15 mm) are at a higher risk of PTB [5, 6]. However, ultrasound scan to measure cervical length is operator-dependent and and may not be avaliable in some clinical settings [6]. Using vaginal biomarkers testing for PTB prediction is an alternative that is reproducible and easily performed. Different biomarkers have been tested such as placental alpha-microglobulin-1 (PAMG-1) [7] or phosphorylated insulin-like growth factor binding protein-1 (pIGFBP-1) [8], but fetal fibronectin (fFN) remains the most promising test [9, 10].

fFN is an extracellular matrix protein present at the decidual- chorionic interface. Disruption of this interface due to inflammation, abruption, or uterine contractions releases fFN into cervicovaginal secretions, which lays the basis for PTB prediction. Qualitative fFN have been extensively studied. In 2013, a systematic review of 5 randomized trials and 15 diagnostic test accuracy studies evaluating cervicovaginal fFN for predicting PTB in women with symptoms of preterm labour reported the pooled estimated sensitivity and specificity of 76.7% and 82.7% for delivery within 7 days of testing. The high negative predictive value (98–100% for delivery within 7 or 14 days of testing [11]. The high NPV (98–100% for delivery within 7 or 14 days of testing) gives clinicians confidence to avoid unnecessary intervention and discharge patients whom are not at risk of PTB [10–12]. A Cochrane review in 2019 [13] also suggested knowledge of fFN results results in symptomatic women may reduce PTB before 37 weeks, compared with controls without such knowledge (21.6% versus 29.2%, risk ratio 0.72, 95% confidence interval 0.52 to 1.01).

Quantitative fFN measurement with the knowledge of the fFN concentration appears to further improve the predictive value. A meta-analysis in 2021, which included 15 studies and 6113 women with or without symptoms of preterm labour, concluded that the pooled sensitivities for thresholds of 10, 50, 200, and 500 ng/ml were 0.78, 0.56, 0.33, and 0.11 and pooled specificities were 0.63, 0.84, 0.96, and 0.99, respectively for detection of PTB at less than 34 weeks of gestation [10]. These results challenges the method of using a single threshold for PTB prediction. fFN concentration helped in decision making for those at highest risk of PTB as well as patient counselling. Current data are largely based on Western populations and data for Chinese women is lacking. A pilot study on qualitative fFN testing on Chinese women has demonstrated negative predictive value of PTB to be 100% accurate [14].

The use of qualitative fFN test for management of patients with symptoms of preterm labour is recommended by various authorities [5, 15–17]. Use of quantitative fFN can further discriminate the risk. The aim of this study is to evaluate the predictive potential of quantitative fFN testing for the prediction of PTB in women with symptoms of preterm labour by a bedside system (PeriLynx) in a Hong Kong population.

## Methods

This was a prospective cohort study conducted between 1st October 2020 and 31st October 2021 in the Department of Obstetrics and Gynaecology of Queen Mary Hospital, Hong Kong. TPTL was defined as symptoms of painful uterine contractions at preterm gestation as well as documented uterine activity on tocogram. Gestational age was assigned on the basis of the last menstrual period (LMP) and confirmed by ultrasonographic measurements in the first trimester. If there is a discrepancy, ultrasonographic estimated gestational age in the first trimester was used.

Inclusion criteria included gestational age between 24 + 0 to 33 + 6 weeks, maternal age of ≥ 18 years old, intact membranes and cervical dilatation of ≤ 3 cm. Women were excluded if they had a prior fFN test performed or a history of tocolysis in the current pregnancy, premature rupture of membranes, multiple pregnancies, prior cervical examination or sexual intercourse within 24 h, moderate or heavy vaginal bleeding, cervical cerclage or placenta praevia. Women were given an information sheet about the study and the use of the fFN test. Written informed consent was signed and patients were enrolled under protocols approved by the Institutional Review Board of The University of Hong Kong/Hospital Authority Hong Kong West Cluster.

During the gynaecological examination, a wet speculum with water as lubricant was introduced into the vagina. Women were asked to cough to look for any leaking of amniotic fluid through the cervical os. If rupture of membranes was diagnosed, women were excluded from the study. fFN testing was done before any digital or transvaginal ultrasound examination. A polyester swab was inserted into the posterior fornix and rotated for 10 s to absorb cervicovaginal secretion. The swab was then placed into the collection tube provided. One aliquot (200 µL) of the sample was analysed with the quantitative Perilynx analyser (Hologic) according to manufacturer’s instructions. The test result was concealed to the patients and the attending physician. Clinical management was at the discretion of the attending physician which included admission for tocolytics, intramuscular corticosteroids and magnesium sulphate as per hospital guidelines. Demographic data were obtained from each patient and extracted from medical records.

Thresholds for fFN were used to determine sensitivity, specificity, PPV, and NPV for spontaneous delivery within 24 h, 48 h, 7 days and 14 days and delivery before 34 and 37 completed weeks of gestation. Results of fFN levels were then grouped into the 6 commonly used predefined incremental categories [10, 11] in the literature (0–9, 10–49, 50–99, 100–199, 200–499, > 500 ng/mL), and the corresponding PTB rates were calculated. The categories were chosen as such to allow analysis of different cut-off thresholds. The threshold of 50 ng/mL was the usual standard threshold to indicate a positive test for qualitative fFN test. The threshold of 500 ng/mL was included because it was the upper limit of the device. The other two thresholds of 100 ng/mL and 200 ng/mL were used following the study protocols by Abbott et al. [12] which already proved that these cutoffs had the greatest predictive power using logistic regression. Data were analysed using Student’s t test for normally distributed continuous variables and the Mann-Whitney U test for non-parametric data. The Chi squared tests were used for dichotomous outcomes. Statistical analysis was done using SPSS version 25.0 statistical software; p < 0.05 was considered statistically significant.

## Results

A total of 49 women with symptoms of TPTL were recruited into the study. One test result was invalid and the patient was excluded from the study, leaving 48 women in the final analysis. Demographic and obstetrics characteristics for the participants were described in Table 1.

**Table 1:** Demographic characteristics

	Total n = 48	PTB n = 10	Term delivery n = 38	p-value
Maternal age (IQR)	35 (5)	34 (6)	35 (5)	0.31
BMI at booking visit, median (IQR)	21.5 (3.6)	20.7(3.8)	21.9(4.6)	0.28
Gestational age of PTB symptoms (weeks),median(IQR)	29.2 (3.8)	30.7	28.9	0.82
Ethnicity				0.11
Chinese	45(94%)	8 (80%)	37 (97%)	
Japanese	1(2%)	0	1 (3%)	
Caucasian	2(4%)	2 (20%)	0	
Obstetric history				0.006
Nulliparous	32(67%)	3 (30%)	29 (76%)	
Multiparous	16(33%)	7(70%)	9(24%)	
History of PTB				0.13
Without a history of PTB	44(92%)	8 (80%)	36(95%)	
With a history of PTB	4(8%)	2 (20%)	2 (5%)	
Mode of delivery				0.57
Vaginal	23(48%)	4 (40%)	19 (50%)	
Caesarean section	25(52%)	6 (60%)	19 (50%)	
Delivery				
< 24 h from testing	4(8%)	4 (40%)	0	
< 48 h from testing	1(2%)	1 (10%)	0	
< 7 days from testing	2(4%)	2 (20%)	0	
< 14 days from testing	0	0	0	
> 14 days from testing	41(86%)	3 (30%)	38 (100%)	
Gestation at delivery				
< 34 weeks	6(13%)	6 (60%)	0	
34–37 weeks	4(8%)	4 (40%)	0	
> 37 weeks	38(79%)	0	38 (100%)	
Tocolysis use				0.001
Yes	21(44%)	9 (90%)	12 (32%)	
No	27(56%)	1 (10%)	26 (68%)	
Corticosteroid use				0.001
Yes	21(44%)	9 (90%)	12 (32%)	
No	27(56%)	1 (10%)	26 (68%)	

BMI: body mass index; IQR: interquartile range; PTB: preterm birth

The median gestational age of women presenting with TPTL was 29.2 weeks’ gestation (IQR 3.8, range 24 weeks to 33 weeks 6 days). In both groups with PTB vs. term delivery, the majority of patients were of Chinese ethicity (80 vs. 97%) with no statistical difference (p = 0.11). There was a statisical difference in obstetric history between the two groups with 29 (76%) patients in term goup being nulliparous compared with 3 (30%) in preterm group (p = 0.006). There was no statistical difference in history of previous preterm birth in preterm and term group (20% vs. 5% p = 0.13). 21 women were given corticosteroids and tocolytics at the discretion of the attending obstetrician. 9 (42.8% of those receiving corticosteroid) had PTB. 12 (57.1% of those receiving corticosteroid) had term delivery after 37 weeks. Overall, 23 (48%) had vaginal delivery; 25 women (52%) had Caesarean delivery. 10 women delivered before 37 + 0 weeks (21% PTB rate) and 6 of them had spontaneous PTB before 34 + 0 weeks. Out of the 10 women with PTB, 6 had caesarean Sect. (5 for preterm labour with malpresentation and 1 for abnormal cardiotocography and previous Caesarean section). 19 women had Caesarean delivery at term with indications such as previous Caesarean section, malpresentation and maternal request.

Concentrations of fFN were obtained for all women with the PeriLynx system. The number of women with results falling within each of the prespecified fFN categories is shown in Table 3. 28 of them had fFN concentration < 10ng/mL whilst 11 of them had fFN concentration > 50ng/mL. For predicting delivery within 48 h to 14 days, using cut-offs of < 50ng/ml and 10ng/ml, the NPV were 97.3% and 100%, indicating that quantitative fFN testing is reliable in ruling out PTB within following 2 weeks.

**Table 2:** Spontaneous preterm birth rate within fetal fibronectin categories

fFN	n	%	PTB		Term births	PTB within 24 h	PTB in 24–48 h	PTB in 2–7 days	PTB in 7–14 days	
(ng/mL)			PTB at < 34 weeks (n, %)	PTB at 34–37 weeks (n, %)	(n, %)	(n, %)	(n, %)	(n, %)	(n, %)	
0–9	28	58	0	2(20%)	26(69%)	0	0	0	0	
10–49	9	19	0	2(20%)	7(18%)	1(2%)	0	0	0	
50–99	5	11	2(20%)	0	3(8%)	0	0	2(4%)	0	
100–199	2	4	0	0	2(5%)	0	0	0	0	
200–499	2	4	2(20%)	0	0	2(4%)	0	0	0	
>/=500	2	4	2(20%)	0	0	1(2%)	1(2%)	0	0	

fFN: Fetal fibronectin, PTB: preterm birth

Note: 3 PTB (< 37w) took place > 14 days of testing

The rate of spontaneous preterm birth at 34 weeks’ gestation was associated increasing concentrations of fFN (Table 2). At the lowest category (0–9 ng/mL), no women had PTB before 34 weeks whereas in the women with fFN ≥ 200 ng/mL, all of them delivered preterm. The PPV for spontaneous PTB (< 37 and < 34 weeks’ gestation) increased from 30 to 40%, 54%, 66–100% and 100% with increasing thresholds (10, 50, 100, 200, and 500 ng/mL respectively) as stated in Table 3.

**Table 3:** The accuracy of fetal fibronectin tests

fFN	Sensitivity	Specificity	PPV	NPV	p value
Preterm delivery < 37w					
> 10ng/ml	0.80	0.68	0.40	0.93	0.0060
> 50 ng/ml	0.60	0.87	0.55	0.89	0.0020
> 100 ng/ml	0.40	0.95	0.67	0.86	0.0030
> 200 ng/ml	0.40	1.00	1.00	0.86	0.0001
> 500 ng/ml	0.20	1.00	1.00	0.83	0.0050
Preterm delivery < 34w					
> 10ng/ml	1.00	0.67	0.30	1.00	0.0020
> 50 ng/ml	1.00	0.88	0.55	1.00	0.0001
> 100 ng/ml	0.67	0.95	0.67	0.95	0.0001
> 200 ng/ml	0.67	1.00	1.00	0.95	0.0001
> 500 ng/ml	0.33	1.00	1.00	0.91	0.0001
Delivery within 48 h					
> 10ng/ml	1.00	0.56	0.25	1.00	0.0010
> 50 ng/ml	0.80	0.84	0.36	0.97	0.0010
> 100 ng/ml	0.80	0.95	0.67	0.98	0.0001
> 200 ng/ml	0.80	1.00	1.00	0.98	0.0001
> 500 ng/ml	0.40	1.00	1.00	0.93	0.0001
Delivery within 7 days					
> 10ng/ml	1.00	0.68	0.35	1.00	0.0010
> 50 ng/ml	0.86	0.88	0.55	0.97	0.0001
> 100 ng/ml	0.57	0.95	0.67	0.93	0.0001
> 200 ng/ml	0.57	1.00	1.00	0.93	0.0001
> 500 ng/ml	0.29	1.00	1.00	0.89	0.0001
Delivery within 14 days					
> 10ng/ml	1.00	0.68	0.35	1.00	0.0010
> 50 ng/ml	0.86	0.88	0.55	0.97	0.0001
> 100 ng/ml	0.57	0.95	0.67	0.93	0.0001
> 200 ng/ml	0.57	1.00	1.00	0.93	0.0001
> 500 ng/ml	0.29	1.00	1.00	0.89	0.0001
PPV: positive predictive value; NPV: negative predictive value					

## Discussion

Our results, in a predominantly Chinese population are consistent with existing literature We show that in women with TPTL; there is a high NPV (100%, 97.3%) with fFN testing allowing the clincian to rule out delivery in the following 2 weeks when using a cut-off of 10ng/ml and < 50ng/ml respectively [10–12]. We also found that 60% of women presenting with TPTL symptoms had fFN levels < 10ng/ml indicating that approximately half of the women present with preterm labour symptoms could avoid unnecessary interventions as were unlikely to go onto to have a PTB.

Previous qualitative fFN testing with a single threshold of 50 ng/ml limited its widespread clinical use due to the low sensitivity and PPV [18]. Quantitative fFN can identify symptomatic women at risk of PTB since the PPV gradually improves with the thresholds indicating the potential application in picking up genuine PTB, overcoming the limitation of qualitative fFN. We find using fFN with 200 ng/ml as cut-off can reliably predict women who delivered within 48 h to 7 days with PPV of 100% and specificity of 100%; as well as PTB before 34 weeks and before 37 weeks of gestation. Suff et al. has demonstrated that the threshold of 200 ng/ml can result in a two-fold increase in the PPV with minimal effect on the NPV; indicating a superior diagnostic accuracy than the 50 ng/ml threshold [19].

Our results have indicated that a cut-off of < 10 ng/ml can be used to rule out PTB. A double blinded study on fFN in some countries may be difficult since fFN testing is already a standard recommendation for suspected PTB [5]. In this study cohort, 21 women were given corticosteroid at the discretion of the attending obstetrician. However 12 of these women, (57%) had a subsequent term delivery. Their fFN results ranged from 1 to 195 ng/ml with 7 women having levels < 10 ng/m. Had the fFN result been made available during decision making, they would not have received the unnecessary corticosteroid injections.

The average length of hopital stay for patients with TPTL in our unit is approximately 2 days, whilst labour ward management is up to 4 days for the use of corticosteroid and tocolytic therapy. Up to USD$3000 could be saved per admission if the woman is stratified as low risk for PTB with fFN testing [20].

Antenatal corticosteroids has been proven to reduce neonatal mortality (9.3 versus 11.9%, RR 0.78, 95% CI 0.70–0.87), perinatal death (13.3 versus 15.6%, RR 0.85, 95% CI 0.77–0.93) and respiratory distress syndrome (10.5 versus 14.8%, RR 0.71, 95% CI 0.65–0.78) [21]. However it may also increase the risk of neonatal hypoglycaemia [22] and mental and behavioural disorders in children [23]. There are also concerns on transient maternal hyperglycemia after corticosteroid use [24]. Indiscriminate use of antenatal corticosteroids should be discouraged. Our results have suggested a cut-off of < 10 ng/ml can be used to rule out PTB and a cut-off of > 200 ng/ml is highly associated with PTB, this gives crucial information to clinicians’ decision making on corticosteroid therapy, tocolysis as well as arrangement of in-utero transfer to facilities with appropriate neonatal care. With the PeriLynx system, the fFN level could be made available within 10 min at the bedside. This modality of point-of-care testing (POCT) enables rapid triage of patients at risk of preterm labour. North et al. has also demonstrated its applicability in rural setting; reflecting the test is simple to perform and could be done in a clinic setting [25].

Patients with results that are not in discriminatory zones of < 10 ng/ml or > 200 ng/ml may benefit from combined testing using transvaginal cervical length measrement (TVCL). Some studies have investigated the use of a combined test to identify pregnancies at risk of PTB. With the use of TVCL measurement, the combined test appeared to perform well in patients with singleton pregnancies [26] and in multiple pregnancies [27, 28]. The NPV of qualitative fFN for delivery within 7 days in singletons and twins with a CL 11–25 mm was 93–100%; giving clinicians reassurance that they could withhold unnecessary corticosteroids and tocolysis [27]. In women with a shortened CL ≤ 25 mm at 22–28 weeks, the risk of PTB < 32 weeks was significantly higher in the positive fFN group (46.2 versus 12.6%) [21]. This indicates there is a role for additional fFN testing in these at-risk women with shortened CL as the fFN test result can modify the risk substantially. Since TVCL requires more staff training and may not be available at small centres or off-hours, fFN testing on patients with suspected PTB may help in setting when TVCL is not avaliable.

fFN testing is useful even in high risk patient groups. A history of PTB is strongly associated with subsequent PTB [27]. 4 patients were identified with a history of PTB before the index pregnancy, 2(50%) had a positive fFN test result of 402ng/ml and 284ng/ml when they presented with abdominal pain at preterm gestation. They had PTB at gestation 26 and 28 weeks respectively. The other two patients had fFN test result of < 10ng/ml and delivered at term.

fFN testing has shown utility for PTB prediction within 14 days [10, 11, 13]. For patients who had PTB < 37 weeks, 3 of them had PTB > 14 days from fFN testing.Therefore, the relevance of fFN testing beyond 14 days needed further evaluation. Patients should be encouraged to seek medical advice again if there is recurrent TPTL symptoms.

The strength of our study would be that all women recruited continued to participate in our study, this is likely due to fFN testing being a simple vaginal swab test performed along with other standard investigations and did not require an additional examination. The attending physician and the women themselves were blinded to the result of fFN test; enabling unbiased management of the women. Regarding the outcomes evaluated, all had been defined in the study protocol at the beginning to evaluate the prediction of the test.

Our pilot study has some limitations. Firstly, this cohort has a small sample size which impact the NPV in particular as the background risk of PTB is relatively low in Hong Kong. Clinical validation of a larger sample will remain necessary in the future. Nevertheless, we show that in a Chinese-dominant cohort our results were consistent with published literature that mainly includes Caucasian subjects [10, 11], supporting its use within differnent ethnicities. The quantitative fFN testing also echoes our previously published data on the use of qualitative fFN testing, confirming its high NPV for patients at risk of PTB [10]. Secondly, this presented results many not be generalizable for multiple pregnancies. Since multiple pregnancy is a risk factor for PTB itself, the use of combined indicators and risk stratification would be more suitable for clinical application.

## Conclusions

Quantitative fFN testing provides different thresholds in addition to the qualitative method (50 ng/mL) to discriminate the risk of PTB in symptomatic women. Depending on the test result, clinicians can discharge patients not at risk of PTB (< 10 ng/ml), perform transvaginal scan for further risk stratification in borderline cases (10–200 ng/ml) and focus on managing at-risk patients with a high (> 200ng/ml) fFN result. The high NPV in our local setting suggests that it could help clinicians rule out PTB in the following 14 days thereby reduce unnecessary interventions and anxiety to the patient. There would also be a significant cost benefit to the health service. There would also be a significant cost benefit to the health service.

## Data Availability

The datasets acquired and/or analysed during the current study are available from the corresponding author on reasonable request.
